# *In vitro* cytoprotective activity of cyanidin 3-glucoside extracts from *Haematocarpus validus* pomace on streptozotocin induced oxidative damage in pancreatic β-cells

**DOI:** 10.1016/j.sjbs.2021.05.065

**Published:** 2021-05-29

**Authors:** Raju Sasikumar, Arub Jyoti Das, Sankar Chandra Deka

**Affiliations:** a Department of Agribusiness Management and Food Technology, North-Eastern Hill University (NEHU), Tura Campus, West Garo Hills, Tura 794002, Meghalaya, India; b Department of Food Engineering and Technology, Tezpur University, Tezpur, Assam, India

**Keywords:** Antidiabetic agent, Anthocyanins, Cyanidin-3-glucoside, Cytoprotective, *Haematocarpus validus*, Oxidative stress, Pancreatic β-cells

## Abstract

Cyanidin-3-glucoside (C3Ghv) compounds were purified and isolated from the anthocyanins extract of *Haematocarpus validus.* C3Ghv were studied for antioxidant and cytoprotective properties on pancreatic β-cells of rat insulinoma cells (RINm5F) against the oxidative stress induced by streptozotocin (STZ). The exposure of RINm5F cells to C3Ghv at concentration of 100 and 200 μg/mL for 24 h reduced 10% and 23% cell viability, respectively, as compared to control cells. The pre-treatment of RINm5F cells with C3Ghv (50 µg/mL) increased the cell viability by 29% as compared to control, on being treated with STZ (10 mM) for 24 h. The pre-treatment of RINm5F cells with C3Ghv (50 µg/mL) for 24 h followed by exposure to STZ (10 mM) for 1 h decreased the generation of reactive oxygen species (ROS) by 57%, generation of nitric oxide by 22.8%, generation of malondialdehyde (MDA) by 32%, the production of p-ERK ½ by 83%, p-JNK by 82.6%, p-MEK by 57%, and p-p38 MAPK by 64%. The C3Ghv treatment also decreased the ratio of apoptotic proteins Bax to Bcl-2 by 61%, and improved the M2 phase of cell cycle by 75% as compared to STZ treated cells. The overall results suggest that C3Ghv protects pancreatic β-cells against oxidative stress-induced apoptosis, thereby implicating the significant role of C3Ghv as an antidiabetic agent.

## Graphical abstract

**Figure:**
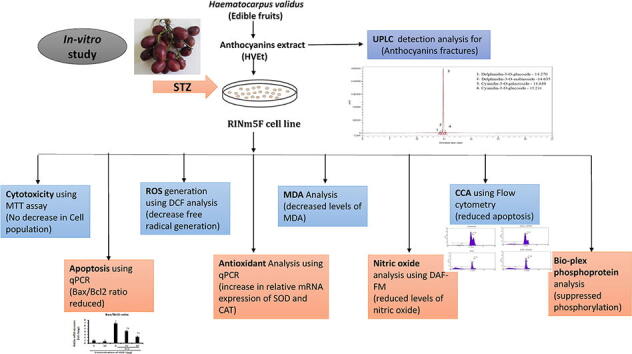

## 1 Introduction

*Haematocarpus validus*, which is commonly known as blood fruit (locally called khoonphal) is a wild edible fruit which belongs to the Menispermaceae family, and widely distributed in Meghalaya and Assam of North-Eastern India (Sasikumar and Deka, 2018, Sasikumar et al., 2020a, Sasikumar et al., 2020b, Sasikumar et al., 2020c, Sasikumar et al., 2020c, Sasikumar et al., 2021a, Sasikumar et al., 2021b, Sasikumar et al., 2021c). It has been utilized in traditional medicine system; and has a copious amount of polyphenols, anthocyanins and flavonoids, which attribute to its antimicrobial, anti-inflammatory and antioxidant properties (Sasikumar et al., 2019a, Sasikumar et al., 2019b, Sasikumar et al., 2019c). The naturally occurring bioactive compounds and antioxidants, which belong to the group of anthocyanins, flavonoids, tannins, phenolic, and alkaloids play a crucial role in alleviating oxidative stress incited apoptosis in cells (Kasote et al., 2020; Monrad et al., 2010). Anthocyanins are a manifold cluster of compounds those are extensively scattered in the plant domain and control a broad spectrum of biological processes due to their anti-allergic, anti-inflammatory, antioxidant, gastro-protective, anti-viral, anti-mutagenic and anti-carcinogenic properties. In our previous study, it has also been reported that the major bioactive volatile compounds in the ethyl acetate extracts of *H. validus* which displayed antioxidant activities were 2-bromotetradecane, tetracosane, heptadecane, eicosane and palmitic acid (Sasikumar et al., 2017, Sasikumar et al., 2020a, Sasikumar et al., 2020b, Sasikumar et al., 2020c, Sasikumar et al., 2020c).

Oxidative stress is characteristic of both Type-1(T1-DM) and Type 2 (T2-DM) diabetes and plays a vital role in hyperglycemia based β-cell dysfunction (Johnson and Luciani, 2010). Increase in the levels of blood glucose results in protein glycation which successively produces Amadori products, Schiff bases, as well as some end products when proteins get exposed to sugars (Ahmed, 2005, Singh et al., 2014). The ROS resulting from this, causes massive loss to pancreatic β-cells as they possess low antioxidant potential (Delmastro and Piganelli, 2011, Wang and Wang, 2017). The ROS induced β-cell death through induction of apoptosis consequently leads to deficiency of insulin secretion (Marrif and Al-Sunousi, 2016, Rojas et al., 2018). Numerous evidences have been documented that apoptosis is the major process involved in death of β-cell in diabetes mellitus (Donath and Halban, 2004, Johnson and Luciani, 2010). Hence, strategies targeted towards apoptotic pathways initiated by oxidative stress have a potential therapeutic application.

A diabetogenic agent, streptozotocin (STZ) causes death of β-cell by DNA damage (Eleazu et al., 2013, Konrad et al., 2001). During STZ metabolism, toxic intermediates such as methyl cations, methyl radicals, ROS and NO radicals are produced (Lenzen, 2008, Murata et al., 1999). It is extensively known that the oxidative stress and apoptosis cause β-cell death in STZ- induced diabetic animals (Gajdosik et al., 1999). The RIN-cell clone RINmF5 is one of the widely used insulin secreting cell lines to study the molecular mechanism of β-cell function and dysfunction (Lenzen, 2008). RINm5F cells have lost ability for glucose stimulated insulin secretion after *in-vitro* culture (Eleazu et al., 2013, Konrad et al., 2001). It has defective glucose recognition apparatus due to a relative lack of the glucose phosphorylating enzyme glukinase (Bharucha et al., 2011). This has demonstrated that continuous cell lines possess many features, including toxic responses that characterize pancreatic islet β-cell linkage (Bargsten, 2004). Hence, RINm5F cell lines have been considered as an ideal model to study β-cell role (Eleazu et al., 2013). Various evidences validated the significance of plant extracts to cure the oxidative stress, mediated apoptosis and death of pancreatic β-cell (Konrad et al., 2001). As products derived from plants have minimal adverse side effects on human system, hence they have a latent application as hypoglycemic medicines. Hence in this study an effort has been made to find the cytoprotective influence of the anthocyanin cyanidin-3-glucoside from *H. validus* against STZ-induced β-cell death in the rat insulinoma cell line (RINm5F).

## 2 Materials and methods

### 2.1 Plant material

*Haematocarpus validus* Bakh.F. Ex Forman (Menispermaceae) ripened fruits were collected from Tura, West Garo Hills, Meghalaya, India in May 2017 and authenticated by Botanical Survey of India with Accession No GUBH18508. The freshly collected fruits had total soluble solid of 17.6 ± 0.2° Brix (at 25 °C), pH of 3.71 ± 0.02 and firmness of 31.5 ± 1.8 N/cm^2^. The collected fruits were stored in refrigerated condition (4 ± 1 °C) until further use.

### 2.2 Chemicals

The analytical chemicals such as methanol, acetonitrile (HPLC grade), 2, 2-diphenyl-1-picrylhydrazyl (DPPH), hydrochloric acid, cyanidin-3-glucoside, iron (III) chloride hexahydrate (FeCl_3_ 6H_2_O), and 2, 4, 6-tripyridyl-s-sriazine (TPTZ) were obtained from Sigma-Aldrich (Steinheim, Germany). All analytical grade chemicals were used for this experimental study.

### 2.3 Samples preparation and anthocyanins extraction

*H. validus* fruit pomace were collected immediately after juice extraction and subjected to drying in a freeze dryer (Alpha 12 LD Plus, Christ Martin, Germany) for 15 h and grounded into powder using a laboratory grinding mill (PEL-DFT-100, Pelican, India) and sieved through 1.5 mm mesh. The extraction of anthocyanins was carried out as per the method of Monrad et al. (2010) with minor alteration. The freeze-dried powder was acidified with aqueous ethanol (50% ethanol; 0.5% HCl, v*/*v) in a ratio of 5:1 (mL/g) solvent:pomace powder and a pH of 3.5 was maintained for better extraction of anthocyanins. Ammonium acetate (25 g) was dissolved in 25 mL of distilled water and added 38 mL of 7 M hydrochloric acid and maintained pH 3.5. This mixed solution (*S*) was transferred to conical flasks and subjected to ultrasonic assisted extraction (UAE) using a probe-type ultrasonicator (Q-1500; Qsonica, Newtown, CHR, USA) with probe diameter of 1/4″ and 5.0 cm height. Sonication was done at 700 W and a frequency of 20 kHz with a maximum amplitude of 240 µm. A cut off time of 3 s was fixed to improve the extraction of anthocyanins (Sasikumar et al., 2019a, Sasikumar et al., 2019b, Sasikumar et al., 2019c). Alternatively, for solvent assisted extraction, the *S* solution was transferred to conical flasks the mouth of which was covered with aluminium foil. Extraction was carried out by continuously shaking at 150 rpm in an orbital incubator shaker (Model: S150, PBI international, Italy) for 24 h at ambient temperature (Sasikumar et al., 2021a). Both the solutions were then filtered through Whatman grade 1 filter paper and the crude anthocyanins extracts were subjected to rotary evaporation (Model: Hei/VAPHL/G3, Heidolph, Germany) at 45 °C for 30 min under vacuum to concentrate and remove ethanol residues from the extracts.

The concentrated crude anthocyanins extracts were purified as per the procedure of Wang (2012) by using SDVB copolymer resins. The resins were activated as per the method recommended by manufacturer and the activated resins (0.6 g) were poured into a 10 mL disposable syringe to make a column. The concentrated crude anthocyanin extracts were dissolved in 100 mL of HPLC grade water and allowed to elute through the resin column. The elution was continued until the anthocyanins adsorbed on resins were saturated, following which the adsorbed resins were washed off with 95% ethanol till the colour of the resins retained back to original. The eluents were then subjected to freeze drying to get fine powder of anthocyanins, which were denoted as SEEt (solvent extraction) and HVEt (ultrasound assisted extraction). These were kept in airtight brown glass container under refrigeration condition (4 °C) to avoid photo-degradation until further use.

### 2.4 Total anthocyanins content (TAC) measurement in HVEt

TAC was analysed as per the procedure described by Chorfa et al. (2016) with minor modifications. The HVEt (1 mg) was dissolved in HCl: MeOH: H_2_O (1:3:16) solution and left to stand for 72 h at 4 °C, after which the absorbance was read at 530 nm. Results were expressed as milligram of cyanidin-3-glucoside equivalent (mg C_3_GE/100 g) of HVEt (db).

### 2.5 Determination of total antioxidant activity of HVEt

DPPH (2, 2-diphenyl-1-picrylhydrazyl), ABTS (2, 2′-azino-bis (3-ethylbenzothiazoline-6-sulfonic acid)) and FRAP (ferric reducing antioxidant power) assays were carried out to determine the total antioxidant activity of HVEt. All the antioxidant activity assays were performed according to the method suggested by Thaipong et al. (2006). The calibration curves for DPPH and ABTS assays were prepared by using the standard Trolox. The standard curve of trolox concentrations (25 to 800 μM) with their respective absorbance was plotted. The final results of antioxidant capacity were expressed as μmol TE/mL HVEt (micromoles of Trolox equivalent per millilitre of db).

### 2.6 Isolation of cyanidin-3-glucoside from HVEt

The isolation of cyanidin-3-glucoside (C3G) from HVEt was carried out as per the procedure of Wang et al. (2014) with slight modification. The C3G were identified and separated from other anthocyanins by using an UPLC system (Waters 2695 series) which consisted of a photo-diode array detector (DAD; Waters 2998, Waters Corporation, USA), a Super C18 column (ACE Excel 5; 5 μm; 125 × 4.6 mm; Aberdeen, Scotland) at column-oven temperature of 25 °C and 10 μL injection loop. Formic acid (10%) and acetonitrile-methanol (85:15, v/v) were mobile phases ‘A’ and ‘B’ respectively. The analysis was carried out at a flow rate 1.0 mL/min by using the following gradient: 0–30 min: 5–12% B, 30–50 min: 12–25% B. The chromatograms of C3G and other anthocyanin were observed at 520 nm and DAD data were generated at 200–600 nm. The standard cyanidin-3-glucoside was dissolved in solvent B (10%) and solvent A (90%) to generate seven-point external standard calibration curve (concentration range was from 1 to 100 mg/L), whose linearity was acceptable (R ^2^ = 0.996). For identification and separation of C3G, 0.1 mg of HVEt was dissolved in the mobile phase (1:10 ratio of solvent A and solvent B) and injected after filtration. The C3G in the HVEt was identified and quantified according to the HPLC retention times (RT) and UV absorbance maximum after comparison with standard. The C3G peak fraction was collected separately. The required quantity C3G for cell line study was collected by performing a number of HPLC analyses. The C3G peak fractions were freeze dried to get purified C3G molecules, and denoted as C3Ghv.

### 2.7 Cells and culture conditions

The rat insulinoma cells (RINm5F) were purchased from National Centre of Cell Sciences (NCCS), Pune, India. The cells were grown as adherent culture in a 5% CO_2_ humidified atmosphere at 37 °C in a medium of RPMI-1640. The amplification was carried out in presence of 10% fetal bovine serum (FBS) along with 10 mg/mL streptomycin and 10,000 U/mL penicillin. RINm5F cells are one of the earliest lines obtained and are initiated from tumours maintained. They are a widely used insulin-secreting cell line and majorly contain insulin and small quantities of glucagon and somatostatin. They were chosen for this study as they are an ideal model to study β-cell apoptosis as they exhibit properties that deviate from the normal condition of glucose transport and/or phosphorylation and possess inappropriate glucose sensitivity (Dereli et al., 1988).

### 2.8 Cytotoxicity of C3Ghv on RINm5F cells

To study the cytotoxicity of C3Ghv on RINm5F cells, the cell viability assay was carried out as per the procedure of Zhang et al. (2012). The cells of RINm5F were seeded onto 96-well plates at a concentration of 2 × 10^4^ RINm5F cells per well and incubated for 24 h. After that, the cells were exposed to different concentrations (0–200 µg/mL) of C3Ghv by adding 10 µL of respective concentration of C3Ghv per well, and incubated for 24 h. The cytotoxicity of C3Ghv was then determined by using MTT reduction assay. The exiting culture media was taken out and 20 μL of MTT (3-(4, 5-dimethylthiazol-2-yl)-2, 5-diphenyl tetrazolium bromide) solution (5 mg/mL in PBS) was added into each well and the cells were incubated further for 6 h. The live cells were converted the MTT compound to formazan crystals due to the action of oxidoreductase enzymes and formazan crystals were precipitated. From each well the supernatants were discarded and then 200 μL of dimethylsulfoxide and 25 μL of glycine buffer (pH 9.5) were added to the wells and mixed properly to dissolve the dark blue formazan crystals. The absorbances of the plates were read at 570 nm in microplate spectrophotometer (Model-Genesys- S 10S UV–Vis, Thermo Fisher, USA) and cell viability was calculated according to Eq. (1).

(1) $$Cellviability\left(\%\right)=\frac{meanODofsample}{meanODvalueofcontrol}\times 100$$

### 2.9 Effect of C3Ghv on STZ-induced cytotoxicity in RINm5F cells

To study the effect of C3Ghv on STZ-induced cytotoxicity in RINm5F cells, the cell viability assay proposed by Zhang et al. (2012) was carried out. The RINm5F cells were seeded onto 96-well plates at a concentration of 2 × 10^4^ cells per well and incubated for 24 h. After that, the cells were exposed to different concentrations (0–50 µg/mL) of C3Ghv by adding 10 µL of respective concentration of C3Ghv per well, and incubated for 24 h. Then 10 µL of 10 mM streptozotocin (STZ) was added onto each of the pre-incubated C3Ghv cells wells and incubated for another 1 h. After that, effect of C3Ghv on STZ-induced cytotoxicity RINm5F cells was determined by using similar MTT reduction assay as mentioned in Section 2.5.

### 2.10 Effect of C3Ghv on ROS generation in STZ treated cells

For measuring STZ-induced ROS levels, the fluorescent dye 2, 7-dichloro fluorescein diacetate (DCFH-DA) was used (Aranda et al., 2013). Briefly, RINm5F cells (2 × 10^4^ per well) suspended in RPMI-1640 medium were pre-treated for 24 h with C3Ghv (0–50 µg/mL) and further subjected to treatment with 10 mM STZ for 1 h at 37 °C. After that 20 μM DCFH-DA was added and further subjected to 30 min incubation at 37 °C. The DCFH-DA gets deacetylated by the cells and results in non-fluorescent DCFH (2′, 7′-dichlorofluorescin) production, which gets converted into fluorescent DCF (2′, 7′-dichlorofluorescein) on oxidation or reaction with ROS. The cells were then washed with phosphate-buffered saline (PBS) and homogenized in 300 μL of 0.1% Triton X-100 (PBS, pH 7.4) by sonication. The homogenates were then incubated at 5 °C for 10 min, and the supernatant was collected by centrifugation, which was used for the assay where the excitation/emission wavelength was 488/510 nm.

### 2.11 Nitric oxide assay of STZ treated cells

To measure nitric oxide production, RINm5F cells were seeded in culture media on a 96-well plate (2 × 10^4^ per well). After an interval of 16 h incubation, treatments of cells were carried out with C3Ghv (0–50 µg/mL) for 24 h followed by treatment with 10 mM STZ and incubation of 1 h. Then, 1 µM solution of 4-amino-5-methylamino-2, 7-difluorofluorescein diacetate was mixed with the incubated sample and again incubation was done for 10 min. A fluorescence spectrophotometer (Model: F4500, Hitachi, Japan) was used to measure the fluorescence at an excitation/emission wavelength of 495/515 nm (Li and Wan, 2015).

### 2.12 Malondialdehyde (MDA) assay of STZ treated cells

For this study, the RINm5F cells (2 × 10^4^ per well) were seeded in respective culture media, which then were allowed to grow for 18 h at 37 °C, followed by C3Ghv treatment (0–50 µg/mL) for 24 h. The medium was then decanted and new medium was added following which the cells were treated with STZ (10 mM) for 1 h. The cells were centrifuged, homogenised and assayed for MDA production using the MDA-586 assay kit (Oxis Research, OR).

### 2.13 RTqPCR analysis of antioxidant and apoptotic genes

The RINm5F cells were seeded onto culture media in a 96-well plate (2 × 10^4^ cells per well), and after 16 h of incubation, the cells were treated with C3Ghv (0–50 µg/mL) for 24 h followed by treatment with 10 mM STZ for 1 h. RNA isolation kit (RNeasy mini kit, Qiagen, Germany) was utilized to extract the total mRNA, which were then transformed to cDNA using cDNA conversion kit (Qiagen, Germany). The cDNA obtained were used to determine the gene expression using RTqPCR system (CFX connect, Biorad cfx connect systems, USA). Experimental conditions for PCR were 95 °C for 5 min, 45 cycles of 95 °C for 20 s, 62 °C for 30 s, and 72 °C for 20 s. The housekeeping gene used was β-actin and was expressed as relative fold change of untreated control. The CFX manager software (BioRad, CA, USA) was used to analyze the qPCR data.

### 2.14 BioPlex phosphoprotein array assay

The phospho-proteins were analysed using Bio-Plex Pro™ Cell Signaling MAPK Panel (Bio-Rad, Hercules, CA) based on multiplex sandwich bead immunoassays. The RINm5F cells were put in a 96-well plate at the rate of 2 × 10^4^ per well. After an interval of 16 h incubation, treatments of cells were carried out with C3Ghv (0–50 µg/mL) for 24 h followed by 10 mM STZ for 1 h. After the treatment, proteins were extracted using the cell factor QG (provided in kit) supplemented along with protease inhibitor cocktail (Thermo scientific, USA), and used for assay based on manufacturer’s instructions. Briefly, 50 μg of protein sample from each experimental condition were incubated overnight in a Bio-Plex Pro™ 96 flat bottom well plate (BioRad, CA, USA) along with fluorescent capturing beads coupled to phosphor-antibodies (phospho-ERK1/2, phospho-JNK, phospho-MAPK, phospho-MEK) and the assay was performed in triplicates. After incubation, automated washing was carried by Bio-Plex Pro™ wash station. After washing, the plates were exposed to detection antibody (biotinylated), followed by streptavidin–phycoerythrin. The samples were then analysed by using Luminex xMAPanalyser (Luminex 100 Milliplex Analyzer, Luminex Corp. USA) and Bio-Plex Manager™ software 6.1 (Bio-Rad, CA, USA). The standard curves determined the phospho-protein concentrations on each plate and results were recorded as mean fluorescence intensities.

### 2.15 Cell cycle analysis using flow cytometry

Cell cycle analysis was carried out by using a fluorescent intercalating agent, propidium iodide (PI) in a flow cytometer (FACS-Calibur, Becton Dickinson, USA). The cells (~1 × 10^3^) were treated with C3Ghv (0–50 µg/mL) for 24 h at 37 °C followed by STZ (10 mM) exposure for 1 h. Then the medium was changed with fresh medium following which it was subjected for analysis. For control, the cells were incubated only in RPMI-1640 medium. The experiments were done in triplicates for each treatment concentration for which approximately 10,000 cells were assessed per sample. Clumps and cell debris were omitted from the analysis by appropriate gating in all cytofluorometric determinations. To determine the cell cycle phases, FL2-H peak area was obtained using a linear scale. CELL-Quest Software (FACS-Calibur, BD Biosciences, USA) was applied to calculate the percentages of cells in the respective cell cycle phases - G1, G2, S and M. The effect of C3Ghv on apoptosis was investigated by the variation in the quantity of sub-G_1_ hypodiploid cells which was computed by using the histograms created by the Cell-Quest computer programs (BD Biosciences, USA)

### 2.16 Statistical analysis

The acquired experimental data were analysed with the help of SPSS Statistics 21.0 software. Analysis of variance (ANOVA) and Duncan’s multiple range test (DMRT) were calculated to signify the test by taking *‘p’* value as ≤ 0.05. Wherever applicable, the analysed data were presented as average ± standard deviation (n = 3).

## 3 Results

### 3.1 Anthocyanins characteristics

**Fig. 1:**
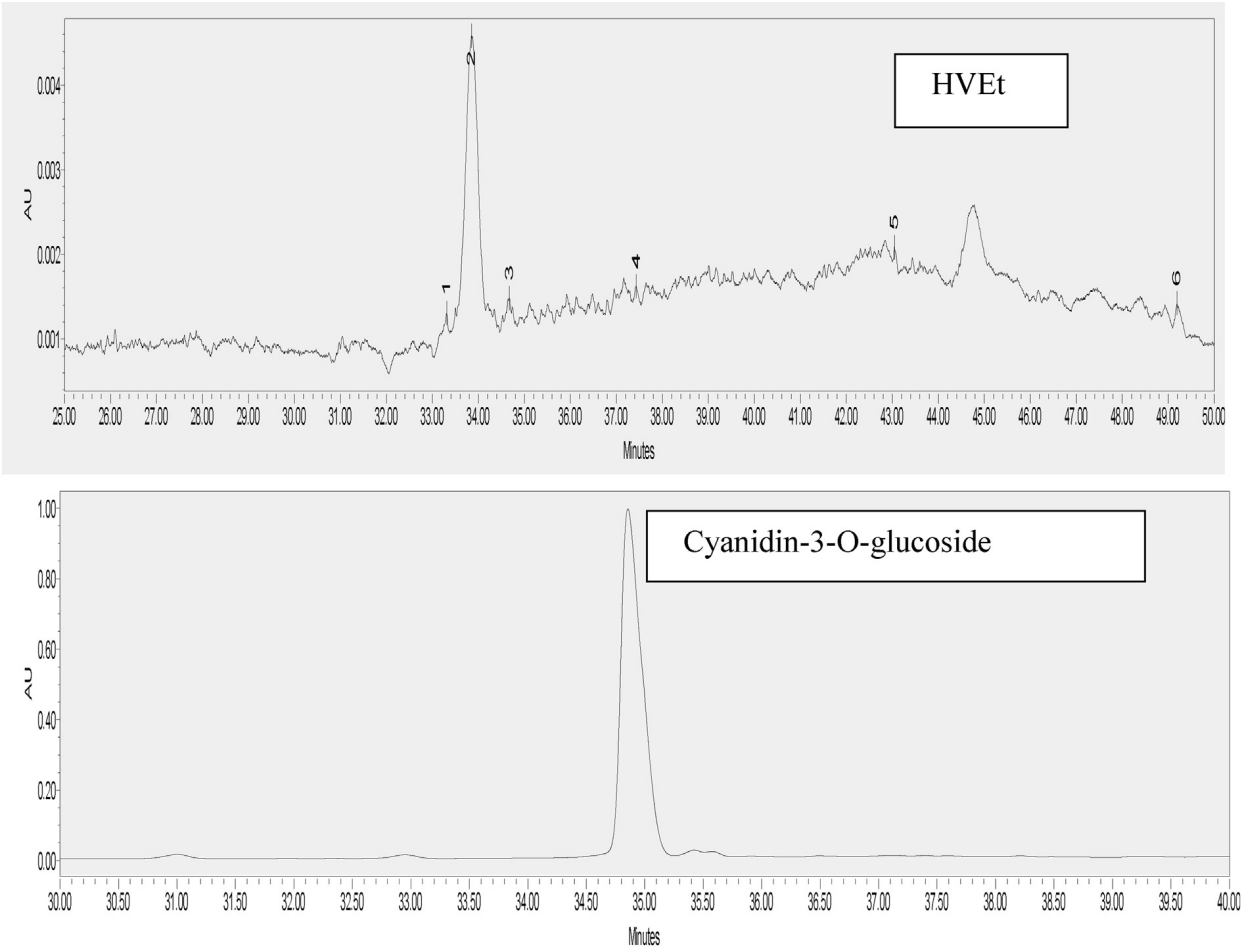
Chromatogram of anthocyanins detected in HVEt by UPLC technique [Peak 1: Cyanidin-3-O-galactoside (RT = 33.31 min), Peak 2: Cyanidin-3-O-glucoside (RT = 33.847 min), Peak 3: Malvidin-3-O-galactoside (RT = 34.673 min), Peak 4 : Peonidin-3-O-arabinoside (RT = 37.431 min), Peak 5 : Malvidin-3-O-glucoside (RT = 43.045 min), Peak 6 : Malvidin-3-O-arabinoside (RT = 49.118 min)]

The total anthocyanins contents (TAC) found in SEEt and HVEt are shown in Table 1. The TAC in HVEt and SEEt found to be 22.78 ± 1.06 and 9.78 ± 0.21 mg C3GE/g respectively. The antioxidant activity of both extract was identified through DPPH, ABTS and FRAP assay. The scavenging activity of HVEt and SEEt obtained from DPPH assay was 890.78 ± 2.40 and 314.77 ± 2.35 µmol TE/g respectively. Whereas, antioxidant activity of HVEt and SEEt obtained from ABTS assay was 639.43 ± 2.13 and 229.11 ± 2.22 µmol TE/g respectively and from FRAP assay was 987.45 ± 2.56 and 338.11 ± 2.55 µmol TE/g respectively. The HVEt further fractionated by UPLC technique (Fig. 1) and anthocyanin fraction such as Cyanidin-3-O-galactoside, Cyanidin-3-O-glucoside, Malvidin-3-O-galactoside, Peonidin-3-O-arabinoside, Malvidin-3-O-glucoside, and Malvidin-3-O-arabinoside were detected (Table 2).

**Table 1:** Total anthocyanins content and antioxidant properties of solvent extraction (SEEt) and ultrasound assisted extraction (HVEt) of anthocyanins extracts from blood fruit.

Parameters	HVEt	SEEt
TAC (mg C_3_GE/g)	22.78 ± 1.06^b^	9.78 ± 0.21^a^
DPPH (µmol TE/g)	890.78 ± 2.40^b^	314.77 ± 2.35^a^
ABTS (µmol TE/g)	639.43 ± 2.13^b^	229.11 ± 2.22 ^a^
FRAP (μmol Fe^2+^/g)	987.45 ± 2.56^b^	338.11 ± 2.55^a^

*Note:* Values represent mean ± SD, n = 3; followed by different superscript letter in a row are significantly different (*p* ≤ 0.05) ^(a-b)^. TAC-Total anthocyanins content, DPPH- 2, 2-diphenyl-1-picrylhydrazyl, ABTS- 2, 2′-azino-bis (3-ethylbenzothiazoline-6-sulphonic acid), FRAP- Ferric reducing antioxidant power, C_3_GE, cyaniding-3-glucoside equivalent, TE- Trolox equivalents

**Table 2:** Anthocyanins characterization from ultrasound assisted extracts (HVEt) detected by UPLC technique.

Peak No	Anthocyanins fractures	Retention time (min)	Concentration (mg/100 g dry weight)
1	Cyanidin-3-O-galactoside	33.31	876.74 ± 1.17^b^
2	Cyanidin-3-O-glucoside	33.847	2961.24 ± 5.78^a^
3	Malvidin-3-O-galactoside	34.673	150.99 ± 1.54^d^
4	Peonidin-3-O-arabinoside	37.431	261.28 ± 1.24^c^
5	Malvidin-3-O-glucoside	43.045	105.11 ± 1.03^e^
6	Malvidin-3-O-arabinoside	49.118	76.23 ± 1.01^f^

Means in columns followed by the different superscripted alphabet (^a-f^) differs significantly (*p* ≤ 0.05); values represent mean ± SD (n = 3).

### 3.2 Effect of C3Ghv on the endurance of RINm5F cells

**Fig. 2:**
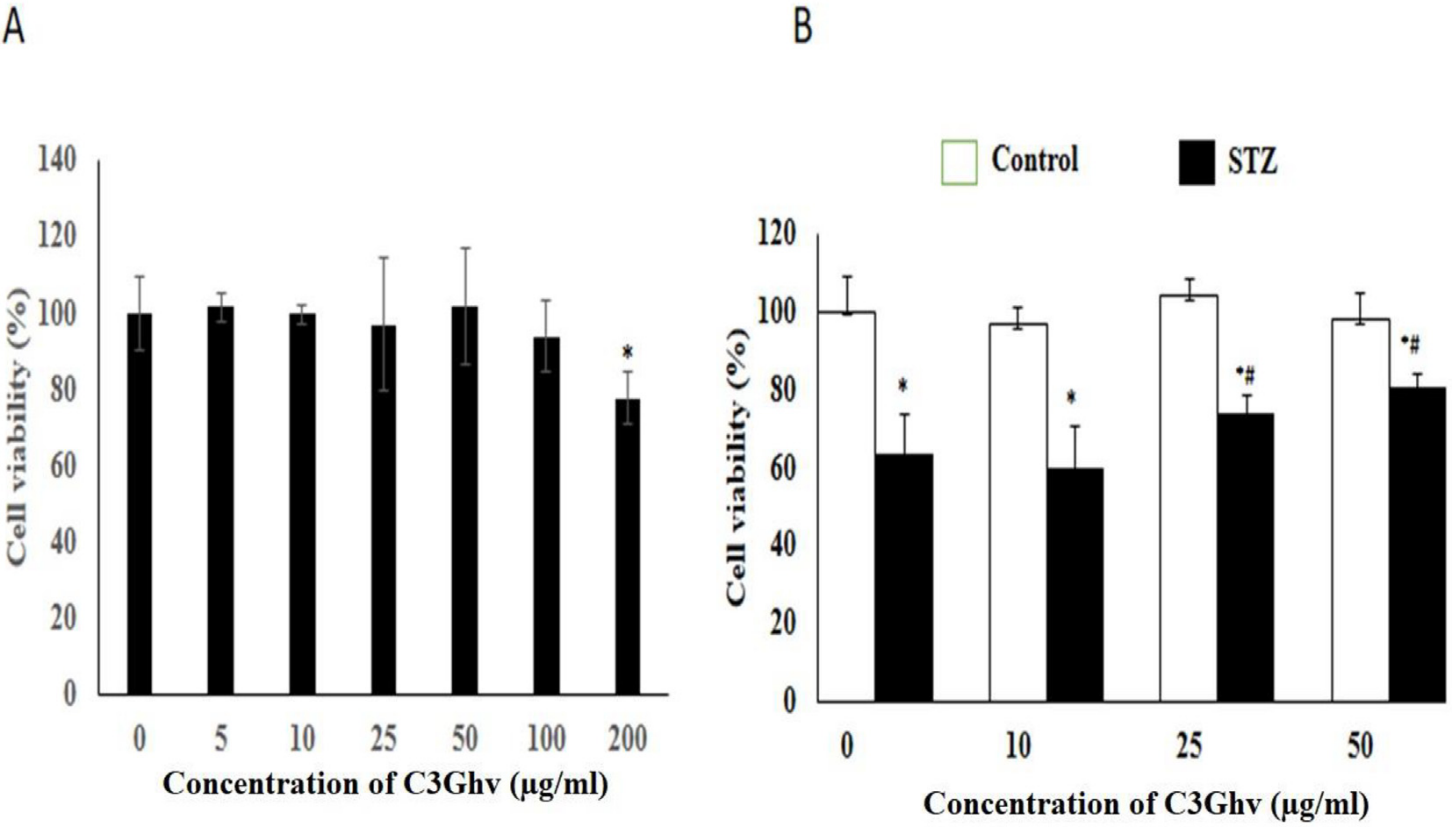
Effect of C3Ghv on oxidative stress-induced toxicity in RINm5F cells. (A) Cytotoxic effect of C3Ghv on RINm5F cells. (B) Cytoprotective effect of C3Ghv against STZ-induced toxicity as assessed by MTT assay (*: differs significantly at *p* < 0.05 compared with the control group. #: differs significantly at *p* < 0.05 compared with the STZ group).

The effect of C3Ghv on the viability RINm5F cells is shown in Fig. 2(A). The pre-treatment of RINm5F cells with different concentrations of C3Ghv (5–200 μg/mL) for 24 h non-significantly (*p* ≤ 0.05) decreased the cell viability. The pre-treatment of the RINm5F cells with C3G at concentration of 100 and 200 μg/mL for 24 h reduced only 10% and 23% cell viability, respectively, as compared to control cells. As the concentration of 100 μg/mL of C3Ghv did not show any adverse effect on RINm5F cells, and therefore chosen for our subsequent studies.

### 3.3 Effect of C3Ghv on STZ-induced cytotoxicity in RINm5F cells

The effect of C3Ghv on the viability against the cytotoxic action of STZ on RINm5F cells is shown in Fig. 2(B). The exposure of RINm5F cells to 10 mM of STZ for 24 h reduced the cell viability by 35% as compared to control. To assess the influence of the C3Ghv against the cytotoxic action of STZ on RIN-5F cells, 10, 25, and 50 µg/mL doses of the C3Ghv were assessed individually against STZ (10 mM) for a period of 24 h. It is manifested from the results depicted in Fig. 2B that C3Ghv is most influential at the concentration of 25–50 µg/mL in protecting RIN-5F cells. It was seen that the doses 25–50 µg/mL of C3Ghv could be either attenuated or suppressed, which resulted retain the viability by 16–29 % against STZ (10 mM) during the period of 24 h as compared to control.

### 3.4 Effect of C3Ghv on the intracellular nitric oxide generation and ROS generation in STZ exposed RINm5F cells

**Fig. 3:**
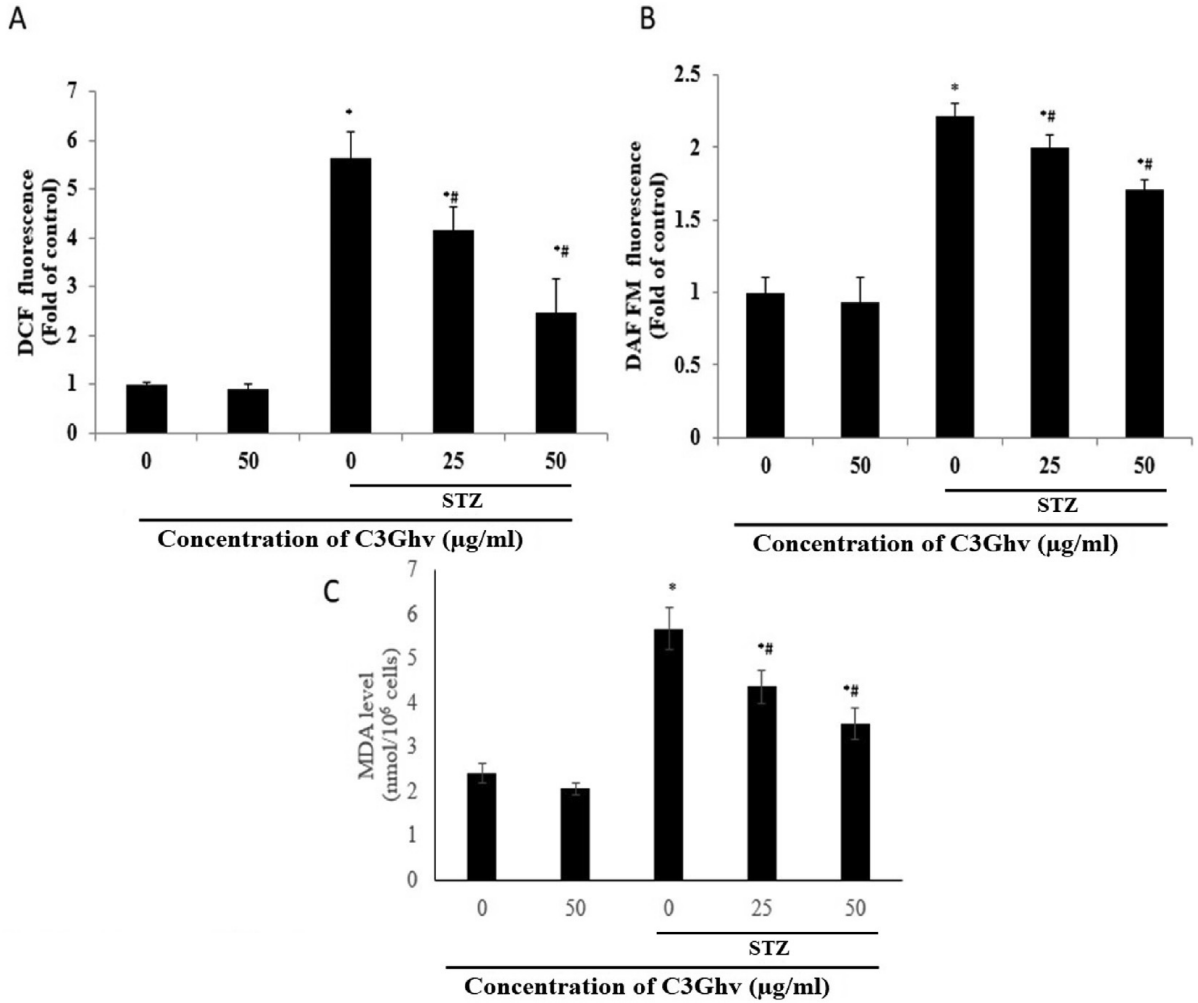
Effect of C3Ghv on the intracellular ROS generation (A), nitrite generation (B) and levels of MDA (C) in STZ-exposed RINm5F cells (*: differs significantly at *p* < 0.05 compared with the control group. #: differs significantly at *p* < 0.05 compared with the STZ group).

To study the molecular mechanism, involve in cytoprotective effect of C3Ghv, the levels of ROS and nitric oxide production was determined using DCF fluorescence. The effect of C3Ghv on the intracellular nitric oxide generation and ROS generation in STZ-exposed RINm5F cells is illustrated in Fig. 3(A-B). The treatment of RINm5F cells with STZ (10 mM) for 1 h increased the ROS generation by 5.64 fold (DFC fluorescence) and nitric oxide generation by 2.25 fold (DAF-FM fluorescence), whereas untreated cells generated only one fold of ROS and nitric oxide. The exposure of RINm5F cells to STZ (10 mM) for 1 h followed by treating with C3Ghv suppressed the generation of ROS and nitric oxide on 25 and 50 μg/mL level of C3Ghv pre-treatment. This suppression of ROS generation was 26% more for 25 μg/mL of C3Ghv and 57% more for 50 μg/mL of C3Ghv, than the ones only treated with STZ (10 mM). Similarly, the suppression of nitric oxide generation was 9.95% more for 25 μg/mL of C3Ghv and 22.8% more for 50 μg/mL of C3Ghv, than the ones only treated with STZ (10 mM).

### 3.5 Effect of C3Ghv on the levels of malonaldehyde (MDA) in STZ-exposed RINm5F cells

The effect of C3Ghv on the intracellular MDA generation in STZ-exposed RINm5F cells is shown in Fig. 3(C). The treatment of RINm5F cells with STZ (10 mM) for 1 h increased the MDA generation to 2.53 nmol/10^6^ cells, whereas untreated cells generated only 2.53 nmol/10^6^ cells. The exposure the RINm5F cells to STZ (10 mM) for 1 h followed by treating with C3Ghv (25–50 µg/mL), suppressed the generation of MDA on 25 and 50 μg/mL of C3Ghv level of pre-treatment. This suppression of MDA generation was 16% more for 25 μg/mL of C3Ghv and 32% more for 50 μg/mL of C3Ghv, than the cells only treated with STZ (10 mM). Thus, the MDA levels in the STZ-exposed cells significantly (*p* ≤ 0.05) decreased with increasing concentrations of C3Ghv.

### 3.6 Effect of C3Ghv on the levels of expression of antioxidants genes in STZ-exposed RINm5F cells as assessed by RTqPCR

**Fig. 4:**
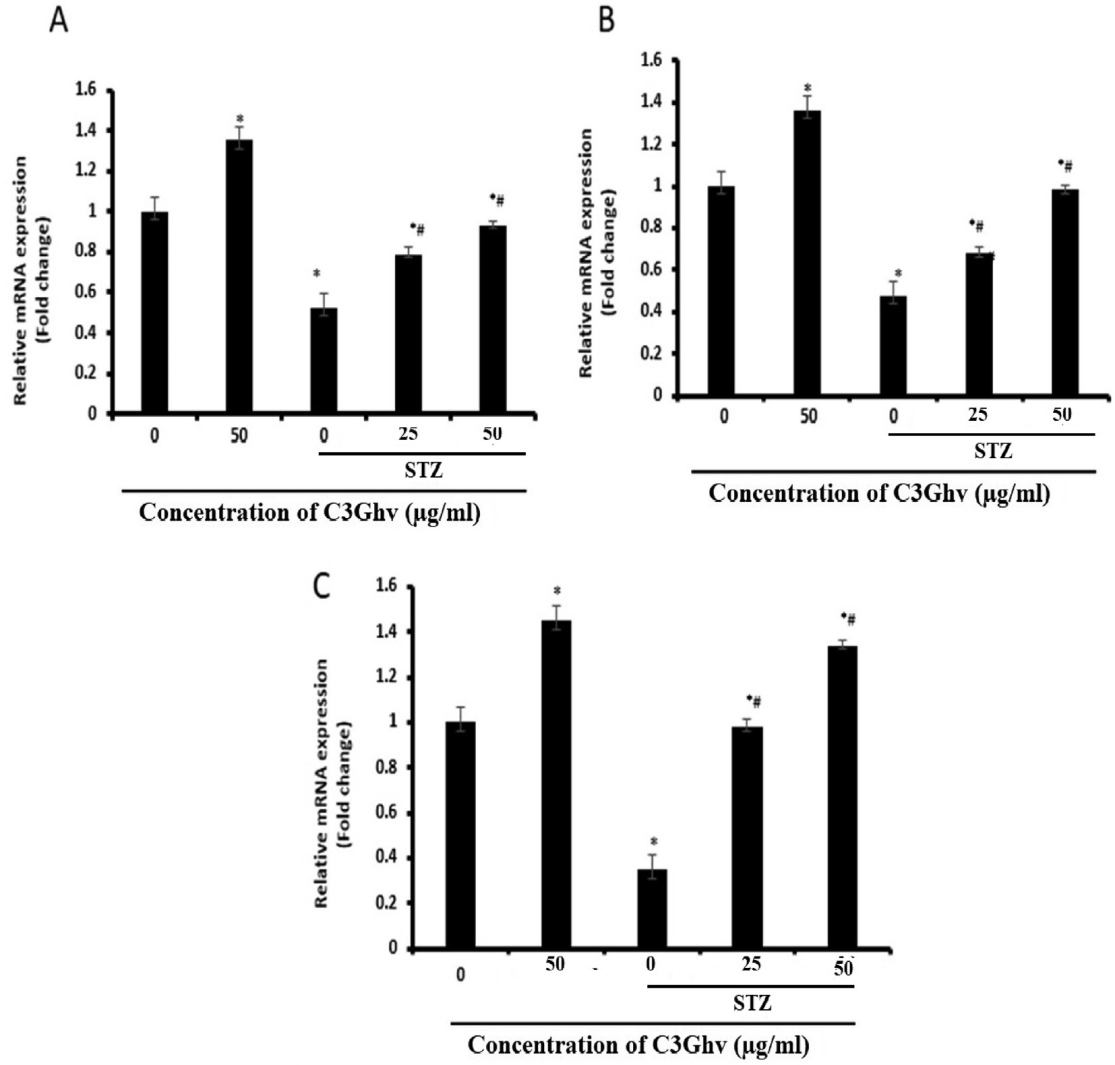
Effect of C3Ghv on the expression of antioxidant genes in STZ-exposed RINm5F cells were measured using qPCR. Transcript levels of (A) SOD (B) CAT and (C) GPx in STZ-exposed RINm5F cells. (*: differs significantly at *p* < 0.05 compared with the control group. #: differs significantly at *p* < 0.05 compared with the STZ group).

The relative mRNA expression of antioxidant genes for SOD, CAT, GPx in STZ-exposed RINm5F cells was performed by qPCR. Effect of C3Ghv on the levels of relative mRNA expression of antioxidant (SOD, CAT, GPx) genes in STZ-exposed RINm5F cells as assessed by RTqPCR is presented in Fig. 4(A). The treatment of RINm5F cells with STZ (10 mM) for 1 h significantly (*p* ≤ 0.05) decreased relative mRNA expression of antioxidant (SOD, CAT, GPx) genes from 52%, 61%, and 74%, respectively than the untreated cells.

### 3.7 Effect of C3Ghv on the levels of phosphoproteins in STZ-exposed RINm5F cells as assessed by multiplex

**Fig. 5:**
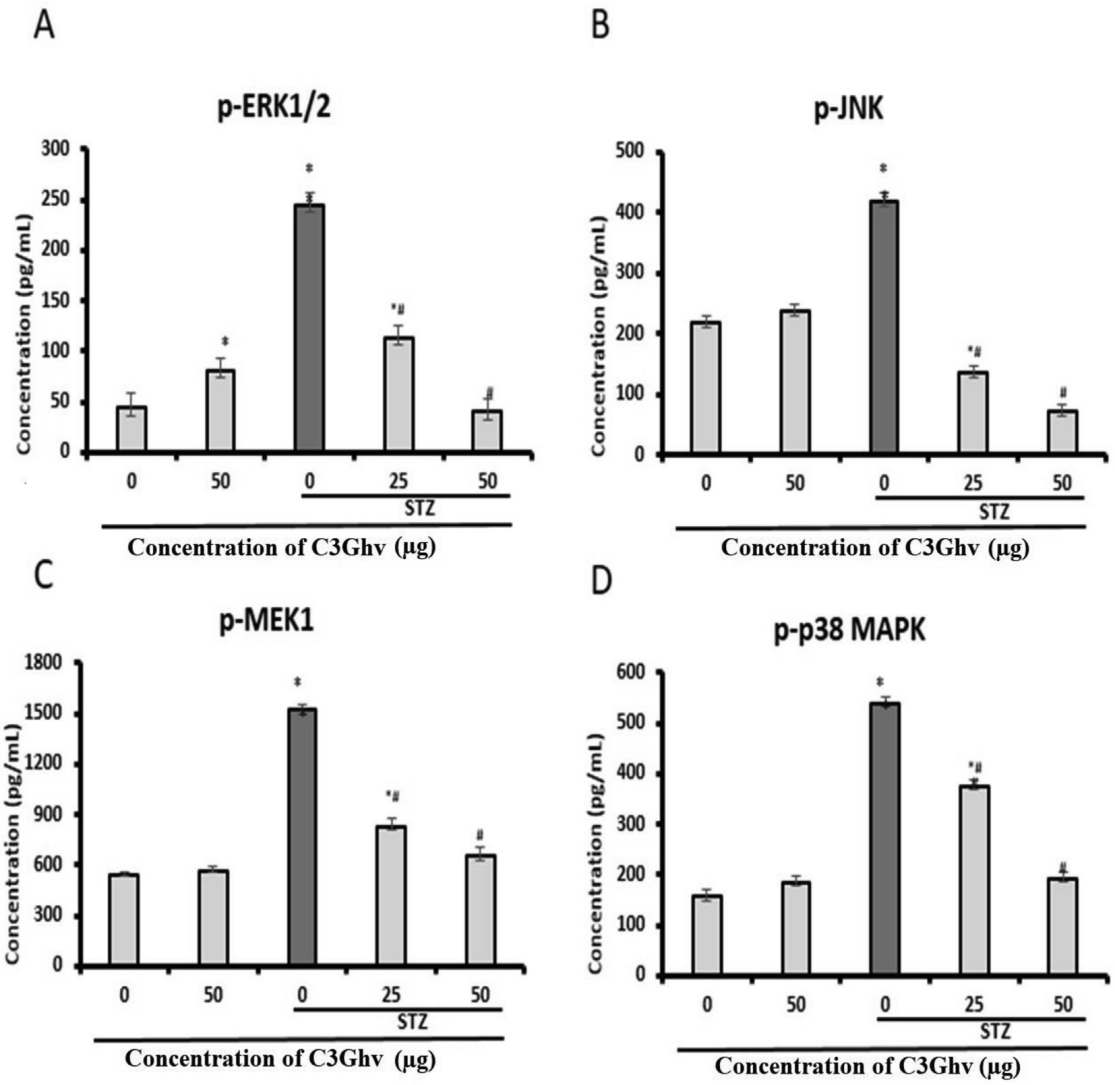
Effect of C3Ghv on upstream phosphokinases viz., (A) pERK1/2 (B) pJNK (C) pMEK1 and (D) pP38 MAPK against STZ-exposed RINm5F cells (*: differs significantly at *p* < 0.05 compared with the control group. #: differs significantly at *p* < 0.05 compared with the STZ group).

The effect of C3Ghv on the levels of phosphoproteins (p-ERK 1/2, p-JNK, p-MEK, and p-p38 MAPK) in STZ-exposed RINm5F cells are shown in Fig. 5(A–D). The treatment of RINm5F cells with STZ (10 mM) for 1 h significantly (*p* ≤ 0.05) increased the production of p-ERK 1/2, p-JNK, p-MEK, and p-p38 MAPK by 245.5, 419, 1521 and 538 pg/mL, respectively. The treatment of RINm5F cells only with 50 µg/mL of C3Ghv decreased the production of p-ERK 1/2, p-JNK, p-MEK and p-p38 MAPK by 81%, 10%, 4%, and 17.5%, respectively, as compared to control cells. The exposure of RINm5F cells to STZ (10 mM) for 1 h followed by treating with 50 µg/mL of C3Ghv significantly decreased the production of p-ERK 1/2, p-JNK, p-MEK, and p-p38 MAPK by 83%, 82.6%, 57%, and 64%, respectively, as compared to STZ treated cells.

### 3.8 Effect of C3Ghv on apoptosis

**Fig. 6:**
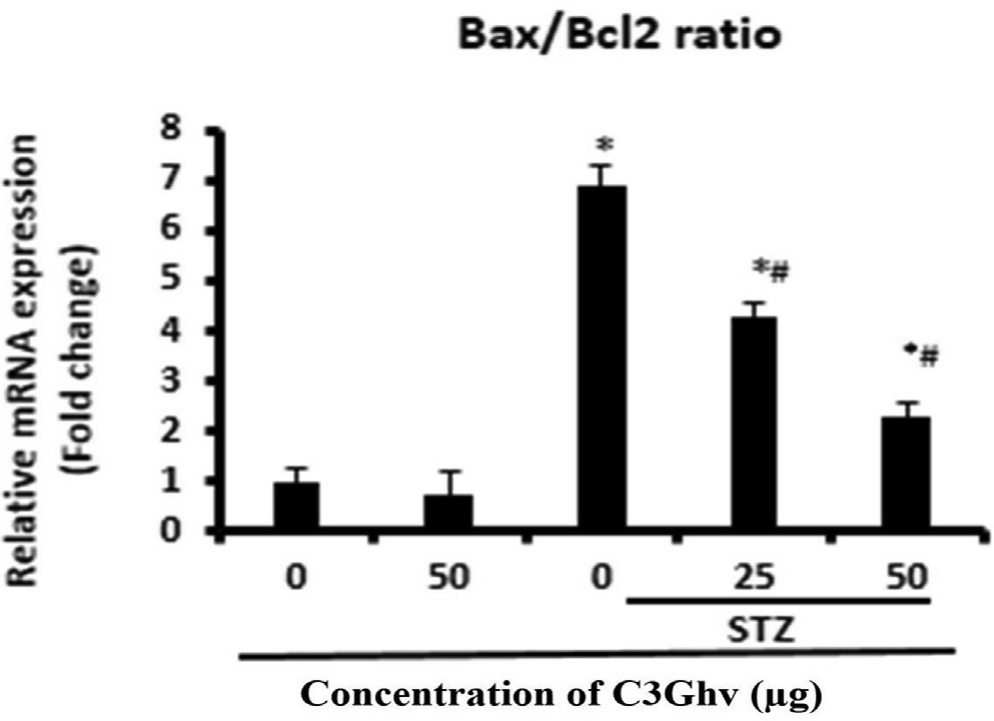
Effect of C3Ghv on the expression of apoptotic genes in STZ-exposed RINm5F cells were measured using RTqPCR (*: differs significantly at *p* < 0.05 compared with the control group. #: differs significantly at *p* < 0.05 compared with the STZ group).

The effect of C3Ghv on the ratio of apoptotic proteins Bax to Bcl-2 is shown in Fig. 6. The treatment of RINm5F cells with only 50 µg/mL of C3Ghv decreased the ratio of apoptotic proteins Bax to Bcl-2 by 45% as compared to control cells. The exposure the RINm5F cells to STZ (10 mM) for 1 h followed by treating with 50 µg/mL of C3Ghv significantly decreased the ratio of apoptotic proteins Bax to Bcl-2 by 61% as compared to STZ treated cells.

### 3.9 Flow cytometric cell cycle analysis of RINm5F cells treated with STZ and/or C3Ghv

**Fig. 7:**
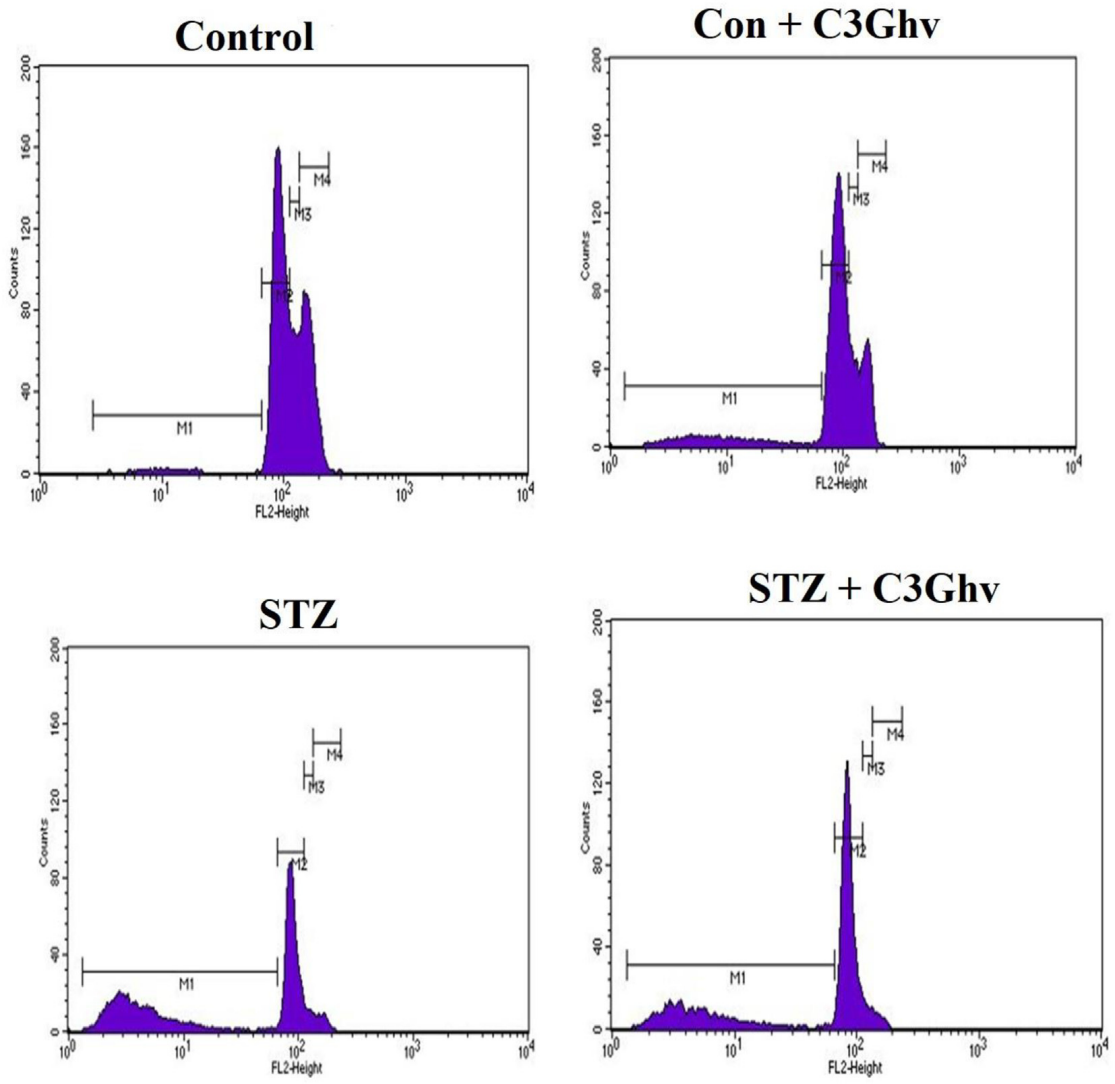
Cell cycle analysis of RINm5F cells treated with STZ and/or C3Ghv measured by flow cytometry.

The effect of C3Ghv on the cell cycle of RINm5F cells treated with STZ is illustrated in Fig. 7. The treatment of RINm5F cells only with 50 µg/mL C3Ghv decreased the M2 phase of cells by 12.5% as compared to control cells. The treatment of RINm5F cells with STZ (10 mM) for 1 h significantly (*p* ≤ 0.05) decreased the M2 phase of cell cycle by 37% then control. The exposure the RINm5F cells to STZ (10 mM) for 1 h followed by treating with 50 µg/mL of C3Ghv significantly improved the M2 phase of cell cycle by 75% as compared to STZ treated cells.

## 4 Discussion

Anthocyanins are hydro-soluble phenolic compounds, which contribute to blood red colouration to the blood fruit and have a number of health and wellness properties. The TAC in HVEt (prepared with ultrasonic treatment) was significantly (*p* ≤ 0.05) higher than in SEEt (prepared with conventional treatment). Thus, the ultrasonic assisted extraction was found to increase the content of TAC by 2.34 fold as compared to solvent assisted extract. The increase in TAC with ultrasonication might be attributed to the cavitation event, which rupture the membrane of anthocyanoplasts and improve cell hydration that results in more dissolution of anthocyanins into the extracted solvent. Singh et al. (2014) reported 203.77 C3GE mg/100 g of pulp in blood collected from Andaman and Nicobar Islands. The biochemical compounds those can inhibit or prevent or delay the oxidation of protein, enzyme, DNA and lipids by free radicals scavenging and oxidative stress diminishing are known as antioxidants. Anthocyanin can prevent oxidation by scavenging free radicals by donating phenolic hydrogen atoms and as metal chelation, and protein binding (Kasote et al., 2015, Rojas et al., 2018, Roux and Blenis, 2004). The antioxidant activities detected by DPPH, ABTS and FRAP assays were also observed to be significantly (*p* ≤ 0.05) higher in HVEt than in SEEt and increased by 2.82, 2.79, and 2.92 fold, respectively. The antioxidant activities viz., DPPH, ABTS and FRAP of ultrasonic assisted extract thus correlated with the TAC of ultrasonic assisted extract. Based on these findings, the ultrasonic assisted anthocyanins extract (HVEt) was considered and chosen for further study. The UPLC detection of anthocyanin fraction of HVEt suggested that, Cyanidin-3-O-glucoside is the prominent form of anthocyanins in HVEt and hence this fraction (C3Ghv) was selected for further study.

The treatment of RINm5F cell against different concentration of C3Ghv revealed that up to the concentration of 100 μg/mL of C3Ghv had no adverse effect on RINm5F cells (Fig. 2A). The obtained results are in agreement with earlier toxicity result reported by Di Nunzio et al. (2017), who stated that at concentration of 120 nM can reduce the cell viability by 93%. After confirming that C3Ghv have no significant effect on RINm5F cells in terms of cell viability, the effect of C3Ghv on STZ-induced cytotoxicity in RINm5F cells was evaluated. The results indicate that C3Ghv had a cytoprotective role in the STZ-exposed RINm5F cells (Fig. 2B). The cytoprotective effect of C3Ghv could be achieved by its glycoside constituents of anthocyanins such as cyanidin-3-O-glucoside, delphinidin-3-O-glucoside and cyanidin-3-O-galactoside, as reported by Wang et al. (2014). A number of cyanindin-3-O-glucoside protective action may arise due to the potent free radical scavenging activity of C3Ghv.

Eizirik et al. (1988) reported that STZ induced β-cell cytotoxic effect can be characterize by increased release of free radicals (Eizirik et al., 1988). The ROS species like peroxynitrite, nitric oxide, superoxide anion etc. are a widely known to cause or responsible for cellular dysfunction and cell death via apoptosis. Increased ROS production results in oxidative damages of cell such as lipid peroxidation, oxidized proteins and DNA damages. On the other hand, nitric oxide (NO) known for its cytotoxic nature reacts with enzymes of the respiratory cycle, which results in interference of DNA synthesis. Moreover, it depletes glutathione by reacting with it, which increases pancreatic β-cell susceptibility to oxidation induced stress (Bedoya et al., 2012). In the progression of diabetes, oxidative stress plays an important role. ROS contribute to diabetes pathogenesis as it leads to cellular membrane damage and alterations in the structural and functional integrity of subcellular organelles (Roy et al., 2017). As a result of naturally low intensity in expression of antioxidant enzyme and their activity in pancreatic islets, there is an increased risk for ROS induced damage than the other tissues (Acharya and Ghaskadbi, 2010, Lenzen et al., 1996). Malondialdehydes (MDA) are also some naturally releasing compounds generates after lipid peroxidation and is cytotoxic in nature and it reacts with free amino groups present in proteins. Prolonged exposure to STZ resulted in enhanced ROS, NO and MDA formation which is implicated by β-cells of pancreatic death (Gille et al., 2002). The results of DCF fluorescence analysis revealed that C3Ghv significantly suppress the generation of intracellular nitric oxide and ROS, when it was treated with STZ exposed RINm5F cells. On the other hand, the results of MDA assay suggested that C3Ghv treatment significantly decrease the MDA formation in STZ exposed RINm5F cells and further decrease the susceptibility of the damage of pancreatic β-cell to oxidative stress.

The results of C3Ghv on the expression of antioxidants genes in STZ-exposed RINm5F cells analyzed by RTqPCR indicated that, the treatment of RINm5F cells with 50 µg/mL of C3Ghv improved the relative mRNA expression of antioxidant genes by 35% for SOD, 36% for CAT, and 45% for GPx as compared to control cells. The exposure of RINm5F cells to STZ (10 mM) for 1 h followed by treating with 50 µg/mL of C3Ghv increased the relative mRNA expression of antioxidant genes by 263% for SOD, 104% for CAT, and 333% for GPx, than the cells only treated with STZ (10 mM). This indicates that C3Ghv treatment led to a substantial raise in relative mRNA expression for SOD, CAT and GPx. Pancreatic β-cells are low in enzymes responsible for antioxidant activity, especially GPx and CAT. In the present study, STZ treatment reduced antioxidant enzyme activity thus making them sensitive to STZ toxicity. Thus, treatment of the cells with C3Ghv might have amplified the activity of antioxidants namely GPx, SOD, CAT and which helps to control the excess ROS formation responsible for apoptosis.

The Ras–Raf–MEK–ERK signalling cascades are responsible for controlling the cell proliferation, hence, these could be mediated by extracellular signal-regulated protein kinases 1 and 2 (ERK1/2). The cellular proliferation and apoptosis are influence by the ERK1/2 translocation to the nucleus and the proteins involved in the cytosolic retention of activated ERK1/2. Cytosolic retention of ERK1/2 denies access to the transcription factor substrates that are responsible for the mitogenic response. The cytosolic ERK1/2 invariably inhibited the survival and proliferative signals in the nucleus and enhance cell death (Šrámek et al., 2016). Recent studies shows that stimulation of proteins such as ERK1/2, JNK, MEK and p-38 promotes β-cell apoptosis through various inflammatory responses (Roux and Blenis, 2004). The current study revealed that the C3Ghv of blood fruit significantly decreased the levels of phosphoproteins in STZ-exposed RINm5F cells. Thus, the decrease in the level of the phosphoproteins indicates the lack of stimulation of pathways associated with apoptosis of β-cell.

Apoptosis is a central process that determines proper development and tissue homeostasis, which is controlled by apoptotic proteins viz., Bax and Bcl-2. Apoptosis of cells is controlled by complex molecular signalling systems wherein proteins encoded by the β-cell CLL/lymphoma 2 (Bcl-2) gene family are chief supervisory components of the apoptotic pathway. The pro-survival protein including Bcl-2 (mitochondrial membrane proteins) can prevent apoptosis. The proapoptotic Bcl-2-associated X protein (Bax) products confined to the cytoskeleton in healthy cells, however, following a death signal, they react predominantly by heterodimerizing with, and inhibiting, the antiapoptotic proteins, thus initiating apoptosis. The cell susceptibility to apoptosis could be determined by the ratio of Bcl-2 to Bax is called pro-apoptotic protein while Bcl-2 is known as an anti-apoptotic protein (Luciani et al., 2013). The communication between Bax and Bcl-2 determined the apoptotic process. Extreme apoptosis of the β-cell in pancreatic are linked to Type 2 (T2DM) and Type 1 (T1DM) types of Diabetes Mellitus (Johnson and Luciani, 2010). STZ (an agent for strong alkalization properties) alkalize DNA present in the pancreatic β-cell, which results in activation of apoptotic signalling pathways. They cause permeabilization of cell membrane and produce the pro-apoptotic effecter- cytochrome *c*, successively facilitate to apoptosis (Bolzán and Bianchi, 2002). It was found that C3Ghv significantly decreased the ratio of apoptotic proteins Bax to Bcl-2 as compared to control cells and helps in the the cell susceptibility to apoptosis (Fig. 6). Hence, it can be conclude that C3Ghv can increase the susceptibility of cells against apoptosis and followed by decrease the risk of occurring diabetes Mellitus.

Flow cytometric cell cycle analysis of RINm5F cells treated with STZ and C3Ghv revealed that, the treatment of RINm5F cells with C3Ghv decreased the M2 phase of cells by 12.5% as compared to control cells. However, the exposure the RINm5F cells to STZ (10 mM) for 1 h followed by treating with C3Ghv significantly improved the M2 phase of cell cycle by 75% as compared to STZ treated cells. There was a decrease in cellular population in the STZ treated cells in M4 and M3 stages, when compared to the STZ untreated cells, thereby indicating a lesser cell population in the synthesis phase. The population of the STZ treated cells also revealed that there was an arrest in proliferation in the G1 phase. Moreover, in case of C3Ghv treatment, there was an increase in the population of cells that progressed into the M and G2 phase, which indicates that STZ treatment hindered cell progression into the S phase. These results demonstrate the specific mechanisms that regulate cell cycle blocking at different checkpoints in response to oxidative stress that is induced by STZ.

## 5 Conclusion

In the present study, the experimental results have the credible evidence that the STZ-induced cytotoxicity in pancreatic β-cells, RINm5F is suppressed by the pre-treatment with the anthocyanin cyaniding-3-glucoside extracted from *H. validus* pomace. No previous reports have provided the mechanism of antidiabetic activity of anthocyanin from *H. validus*, and hence this is a novel study which explains the maintenance of pancreatic insulin secretion capacity against oxidative stress. Thus, treatment with cyaniding-3-glucoside could potentially yield therapeutic benefits in diabetes by preserving pancreatic β-cells from apoptosis.

## Declaration of Competing Interest

The authors declare that they have no known competing financial interests or personal relationships that could have appeared to influence the work reported in this paper.
